# Racial and Ethnic Differences in Time to Completion of Academic Enrichment Program Applications

**DOI:** 10.7759/cureus.60054

**Published:** 2024-05-10

**Authors:** Kristian V Jones, Anissa Chitwanga, Qian Qiu, Aspen Avery, Darya Yemets, Carolyn Theard-Griggs, Chelsea Hicks, keith Hullenaar, Monica S Vavilala, Marie A Theard

**Affiliations:** 1 School of Social Work, University of Washington, Seattle, USA; 2 School of Social Work, University of Illinois, Chicago, USA; 3 Harborview Injury Prevention & Research Center, University of Washington School of Medicine, Seattle, USA; 4 Education, National Louis University, Chicago, USA; 5 Pediatrics, University of Washington, Seattle, USA; 6 Epidemiology, University of Washington, Seattle, USA; 7 Anesthesiology and Pain Medicine, University of Washington, Seattle, USA

**Keywords:** academic programs, enrichment, equity, education, injury prevention

## Abstract

Introduction: Diversity in healthcare and research is integral to serving our increasingly diverse population. Access to academic enrichment programs, an important pathway to science, technology, engineering, and mathematics (STEM) careers promotes educational attainment through academic preparation and increased interest, useful strategies for improving diverse representation in higher learning. Given this important pathway to STEM fields, attention to equity in enrichment programs admissions is as important as the increasing focus on mitigating racial/ethnic disparities in undergraduate and graduate admissions.

Methods: In a retrospective cohort study at the University of Washington, we used descriptive and Chi-Square statistics to compare a hybrid competitive summer application program with stipend with an asynchronous first-come, first-served enrollment program in injury and violence prevention research. The three main outcomes were: 1) time to application, measured by number of days to apply/enroll after application or enrollment period start date, 2) percentage of application/enrollment period, measured by when application or enrollment occurred in relation to the total application or enrollment period, and 3) differences in Black, Hispanic, and Native American applicants and enrollees.

Results: In a study examining two injury and violence prevention programs, which reached educational institutions including Historically Black Colleges and Universities (HBCU) and Tribal Colleges: 1) Applicants were 9.6% and 6.4% Black (application vs enrollment programs; p<0.0001), 0.4% and 0% Native American to the application and enrollment programs, and 9.1% and 10.3% Hispanic (application vs enrollment programs; p=0.6), 2) Across all racial and ethnic groups, students applied later (last 15% percent of application period) in the competitive application program than to the first-come first-served enrollment program in which students enrolled throughout the enrollment period, and 3) Across both program types, there were racial and ethnic differences in time to application and enrollment start and completion.

Conclusion: Findings show that free enrollment programs alone do not incentivize educational attainment for all groups and that application rolling admissions processes may not equally promote racial and ethnic diversity for all groups.

## Introduction

The importance of diversity in our biomedical workforce is clear. Diversity in public health research and other health-related professions contributes to increased recruitment and participation of underrepresented groups in clinical trials, and improved patient satisfaction and outcomes [1-3]. However, despite, the rapid growth in the science, technology, engineering, and mathematics (STEM) workforce over the past few decades, Black and Hispanic/Latino personnel remain underrepresented in these fields (9% Black and 8% Hispanic) [4]. Affirmative action bans in secondary education in the 1990s have contributed to the slow growth of racially and ethnically diverse candidates in the science fields [5-8] and the most recent United States affirmative action ban on race-conscious college admissions may negatively impact needed STEM workforce diversity. Examination of the impact of affirmative action bans in six states found that the greatest losses in underrepresented minority (URM - defined as Black, Hispanic, and American Indian/Alaskan Native graduate) enrollment were in science and engineering fields [9]. Important impediments to the progress of URM toward STEM careers include a lack of familiarity with these professions, mentoring, and resources; inadequate academic preparation; and discrimination. Attention to correcting the underrepresentation of members of different racial and ethnic groups in STEM fields will require circumventing bans in part by programming centered on mitigating these barriers to support students. 

Researchers have identified that one way to support students, particularly underrepresented students in higher education, is through academic enrichment programs [10]. An academic enrichment program is a supplementary educational experience designed to enhance students’ academic experiences and challenge them academically [11-14]. In the case of STEM fields, these programs serve to stimulate interest in and increase familiarity with healthcare and public health research [14]. Identity theory posits that STEM enrichment programs are an important social structure for development of a science identity [14]. Results from a national panel study of undergraduate science majors reveals that science identity salience together with college grade point average (GPA) and research experience act as mediators of STEM enrichment program effects on graduate school matriculation [14]. Mentorship, often a component of enrichment programs, is integral to cultivating this science identity particularly for women and members of underrepresented races/ethnicities who may feel excluded in disciplines constructed as masculine and/or White [15]. Students who participate in educational enrichment programs are more likely than students with similar academic backgrounds to sustain an interest in STEM, perform better academically, complete STEM degrees and attend graduate school [10,16,17]. Targeting students from underrepresented groups offers an opportunity to increase the representation of members of these groups in STEM careers [14]. 

While much of the focus on racial differences in higher education admissions in the United States focuses on undergraduate and graduate admissions [18], recent data suggests that social justice selection approaches are critical in supporting access to higher education enrichment programs [19]. This is important to consider as research illustrates students can gain awareness of potential career paths, gain necessary training for career development, and access to mentorship from people in their desired career path in higher education internship programs [15-22] but not all groups have equal access to educational programs and/or attainment [23]. The purpose of this study was to compare time to application and enrollment completion by applicant and enrollee race and ethnicity between a competitive research enrichment program with application, stipend, and deadline versus a free asynchronous first come first served enrollment certificate enrichment program.

## Materials and methods

Study design 

In a retrospective cohort study, we compared our experience with two higher education enrichment programs in pediatric injury and violence prevention funded by the National Institutes of Child Health and Human Development (NICHD): 1) A competitive application program which is an eight-week summer research education training program and 2) An enrollment certificate program which is an online introductory education training program in which students have eight-week access to complete a four-week curriculum. We examined data from the competitive application program between 2019 - 2023 and data from the enrollment program between 2022 - 2023. The University of Washington determined that this work of 2650 learners was exempt from human subjects' research. 

General marketing strategies for programs 

For both the application and enrollment programs, we advertised on the Harborview Injury Prevention and Research Center (HIPRC) website (www.hiprc.org), on social media platforms (X- formerly known as Twitter, Facebook, LinkedIn, and Instagram), Handshake (https://joinhandshake.com/), and via direct emails. We responded to student inquiries through an email portal and fielded inquiries via Handshake Messages. Intentional outreach to Tribal Colleges and Historically Black Colleges and Universities occurred through direct email communications with known contacts. Program advertising began two months prior to application and enrollment opening. The application program also utilized two months of paid marketing on Google Ads as a new strategy in 2023. 

Summary of application and enrollment processes 

Two thousand four hundred ninety-four applicants and 156 enrollees completed structured online application and enrollment forms for respective programs that included self-report demographics such as race/ethnicity, and gender, as required for NIH reporting, and academic questions such as grade point average and research interests. Application/enrollment forms were completed through Google Forms from 2019 - 2022 and through the University of Washington’s Research Electronic Data Capture (REDCap) database in 2023 [24]. Application/enrollment forms were downloaded and stored on a secure server for review by program staff. We used the National Institutes of Health race and ethnicity categories (Underrepresented Racial and Ethnic Groups | Diversity in Extramural Programs (nih.gov), where race and ethnicity were optional data fields for applicants to complete. For the application program, accepted students were informed by email three months post application close date. For the enrollment program, 156 enrollees were notified by email the week of the enrollment close date and were also sent email reminders of their enrollment in the program multiple times before the program start date. 

Overview of application and enrollment programs 

The application program provides a paid eight-week summer research and didactic experience and mentorship for undergraduate, graduate, and first-year medical students who are interested in injury and violence prevention. Participants are selected based on a combination of scholastic thresholds, alignment of personal statements with the mission of the program. When selected, participants are matched to research projects and partnered with health sciences faculty from across the University of Washington and work alongside peers and health professionals from an array of disciplines. Past research areas include elucidating the risk factors and causes of injuries, injury and violence prevention strategies, acute and chronic care of injured patients, outcomes from trauma, and interventions to return the injured individual to their full potential. Students present their research at an end of the program symposium and have opportunities for continued research engagement and publication with mentors. Applicant eligibility requirements include being enrolled in a higher education program (undergraduate, graduate, or medical school program), able to work in the United States, and attending the full duration of the eight-week program. From 2019 - 2021, there was a minimum GPA requirement of 3.5. From 2022 - 2023, the minimum GPA requirement was lowered to 3.0 as prior evaluation showed that lowering the screening GPA does not affect the final cohort GPA of accepted students or change diversity characteristics [19]. Program staff select students based on alignment of interest with program objectives which is evaluated from multiple application questions. On average, over 450 students apply each year and compete for 20 positions. From 2019 - 2023, this program was conducted in-person (2019), virtually (2020 - 2022; due to COVID-19 pandemic), and in hybrid (2023) learning environments. Notification of acceptance occurred on a prespecified date to all students accepted into the program. 

The enrollment program is a free virtual asynchronous four-week (late spring of academic year) enrollment certificate program. There are no requirements aside from being enrolled or recently graduated from a higher educational program, and up to 100 students can enroll. Students are introduced to a variety of injury and violence prevention topics and receive eight-week exclusive access to a four-week curriculum including injury prevention education modules and lectures. The curriculum was offered by leading injury and violence prevention experts. Students who complete the program receive a Certificate in Injury and Violence Prevention. On average, 78 students enroll in the program per year. Enrollment notification to students occurred on a first come first served basis. 

Race and ethnicity categories 

For individual applicants, we categorized self-reported race as White, African American, Native American, Asian/Asian American, Native Hawaiian/ Pacific Islander, Middle Eastern, Mixed, and “My race is not represented”, and “prefer not to say”. For individual applicants, self-reported ethnicity was categorized as Hispanic, non-Hispanic, and unknown. We included the "prefer not to say" group because they represented 4% of the applicant pool and 3.2% of the enrollees, equivalent or larger than some of the other racial/ethnic groups. We considered examining URM applicants collectively as an underrepresented in medicine group but did not because of lack of representation of Native American students in the enrollment program. 

Outcomes 

The two main outcomes were time to application and time to enrollment measured by: 1) number of days to apply/enroll after application or enrollment period start date and 2) percent of application and enrollment period calculated by dividing number of days to apply/enroll by total number of days in application/enrollment period. 

Statistical analysis 

We used descriptive statistics counts (n), percent (%), mean (standard deviation (SD)), and median (interquartile range (IQR)) to characterize each program cohort by and across years. We used Chi-Square to compare programs for Black vs non-Black and Hispanic vs non-Hispanic applicants and enrollees. 

Since start and end dates for application and enrollment periods varied over the years and across the two programs, application period (in days) was calculated for the application program from 2019 to 2023 and enrollment period (in days) was calculated for the enrollment program from 2022 to 2023. Application and enrollment days were defined as the number of days from application and enrollment period opening to day when students applied or enrolled, respectively. 

To calibrate the variation of application and enrollment period across years, we computed percent of application period as (application day/application period)*100 for the application program, and percent of enrollment period as (enrollment day/enrollment period)*100 for the enrollment program. Student applicant and enrollment characteristics were reported as count (percentage) for categorical data and mean (SD) for continuous factors. Median (IQR) application and enrollment day and median (IQR) percent of application and enrollment period are displayed by student self-reported race and ethnicity. Graphs of cumulative percentage over 1-100% application and enrollment period were used to examine time to application and enrollment for race and ethnicity groups as defined above. Statistical analysis was conducted using Stata/MP 15.1 (StataCorp, College Station, TX, USA). 

## Results

Table 1 details applicant demographics for the application program. Over the course of five years, the application program received 2,494 applications of which applicants were mostly female (74.6%) and self-identified as White (34.8%), Asian (35.8%), Black (9.6%), or Native American (0.4%). Approximately 21.9% of applicants identified as an underrepresented minority, including students who self-reported as Hispanic, African American, Native American, or Native Hawaiian/Pacifica Islander. Students who preferred not to report on race or ethnicity or students who self-reported as mixed race without providing race or ethnicity details were not considered an underrepresented minority. Most applicants were enrolled in an undergraduate program (90.5%), with 21.8% of applicants identifying as a first-generation student.

**Table 1:** Application Program (Hybrid Summer Undergraduate Research Program) Applicant Characteristics (2019 to 2023). Note. *Mutually exclusive race categories. **National Institutes of Health Underrepresented Minority (URM): students who ever self-reported as Hispanic, Black, Native American, or Native Hawaiian/Pacific Islander (calculated from non-mutually exclusive race categories). Students who preferred not to report on race or ethnicity, race or ethnicity was unknown or not represented, or students who self-reported as mixed race without providing race or ethnicity details were not considered an underrepresented minority (Underrepresented Racial and Ethnic Groups, n.d).

	Combined	2019	2020	2021	2022	2023
Factor	n=2494	n=452	n=452	n=732	n=428	n=430
Application start date		11/1/2018	11/1/2019	11/19/2020	11/15/2021	11/14/2022
Application end date		1/15/2019	1/19/2020	2/1/2021	2/11/2022	2/10/2023
Application period (days)		76	80	75	89	89
Apply day, median (IQR)	71 (57, 76)	73 (64, 75)	74 (67, 76)	63 (51, 73)	72 (60, 79)	79 (58, 88)
Percent of application period, median (IQR)	88.2 (73.0, 97.3)	96.1 (83.6, 98.7)	92.5 (83.8, 95.0)	84.0 (67.3, 97.3)	80.9 (67.4, 88.8)	88.8 (65.2, 98.9)
Gender, n (%)						
Female	1860 (74.6)	329 (72.8)	324 (71.7)	580 (79.2)	318 (74.3)	309 (71.9)
Male	592 (23.7)	110 (24.3)	122 (27.0)	150 (20.5)	100 (23.4)	110 (25.6)
Unknown	42 (1.7)	13 (2.9)	6 (1.3)	2 (0.3)	10 (2.3)	11 (2.6)
Hispanic, n (%)						
Yes	228 (9.1)	35 (7.7)	43 (9.5)	70 (9.6)	34 (7.9)	46 (10.7)
No	2228 (89.3)	397 (87.8)	397 (87.8)	662 (90.4)	394 (92.1)	378 (87.9)
Unknown	38 (1.5)	20 (4.4)	12 (2.7)	0 (0.0)	0 (0.0)	6 (1.4)
Race,* n (%)						
White	869 (34.8)	165 (36.5)	153 (33.8)	271 (37.0)	141 (32.9)	139 (32.3)
African American	240 (9.6)	46 (10.2)	35 (7.7)	70 (9.6)	52 (12.1)	37 (8.6)
Native American	10 (0.4)	7 (1.5)	1 (0.2)	0 (0.0)	0 (0.0)	2 (0.5)
Asian/Asian American	893 (35.8)	137 (30.3)	171 (37.8)	251 (34.3)	175 (40.9)	159 (37.0)
Native Hawaiian/Other Pacific Islander	3 (0.1)	0 (0.0)	1 (0.2)	0 (0.0)	0 (0.0)	2 (0.5)
Middle Eastern	60 (2.4)	8 (1.8)	9 (2.0)	19 (2.6)	8 (1.9)	16 (3.7)
Mixed	279 (11.2)	54 (11.9)	53 (11.7)	90 (12.3)	39 (9.1)	43 (10.0)
My race is not represented	39 (1.6)	0 (0.0)	12 (2.7)	13 (1.8)	5 (1.2)	9 (2.1)
Prefer not to say/Missing	101 (4.0)	35 (7.7)	17 (3.8)	18 (2.5)	8 (1.9)	23 (5.3)
Underrepresented minority,** n (%)						
Yes	545 (21.9)	99 (21.9)	94 (20.8)	162 (22.1)	95 (22.2)	95 (22.1)
No	1859 (74.5)	327 (72.3)	340 (75.2)	550 (75.1)	327 (76.4)	315 (73.3)
Unknown	90 (3.6)	26 (5.8)	18 (4.0)	20 (2.7)	6 (1.4)	20 (4.7)
1st generation, n (%)						
Yes	544 (21.8)	88 (19.5)	108 (23.9)	158 (21.6)	94 (22.0)	96 (22.3)
No	1908 (76.5)	357 (79.0)	343 (75.9)	561 (76.6)	323 (75.5)	324 (75.3)
Unknown	42 (1.7)	7 (1.5)	1 (0.2)	13 (1.8)	11 (2.6)	10 (2.3)
GPA, mean (SD)	3.7 (0.4)	3.6 (0.4)	3.6 (0.6)	3.7 (0.3)	3.7 (0.2)	3.7 (0.3)
School year, n (%)						
Four-year program	2257 (90.5)	417 (92.3)	416 (92.0)	667 (91.1)	390 (91.1)	367 (85.3)
Two-year Community College	25 (1.0)	4 (0.9)	5 (1.1)	8 (1.1)	4 (0.9)	4 (0.9)
Medical School	87 (3.5)	15 (3.3)	10 (2.2)	12 (1.6)	17 (4.0)	33 (7.7)
Graduate School	100 (4.0)	12 (2.7)	19 (4.2)	35 (4.8)	11 (2.6)	23 (5.3)
Post-Baccalaureate	25 (1.0)	4 (0.9)	2 (0.4)	10 (1.4)	6 (1.4)	3 (0.7)

Table 2 details demographics for the enrolment program. Over the course of two years, the enrolment program enrolled 156 students and enrolees were mostly female (80.8%) and self-identified as White (32.1%), Asian (34.6%), Black (6.4%), or Native American (0%). Approximately 28.2% of enrolled identified as underrepresented minority, including Hispanic (10.3%) and African American (16.0%). Most were enrolled in an undergraduate program (62.8%), with approximately 30.1% of applicants identifying as a first-generation student. 

**Table 2:** Enrollment Program (Asynchronous Virtual Program) Enrollee Characteristics (2022 to 2023). *Mutually exclusive race categories. **National Institutes of Health Underrepresented Minority (URM): students who ever self-reported as Hispanic, Black, Native American, or Native Hawaiian/Pacific Islander (calculated from non-mutually exclusive race categories). Students who preferred not to report on race or ethnicity, race or ethnicity was unknown or not represented, or students who self-reported as mixed race without providing race or ethnicity details were not considered an underrepresented minority (Underrepresented Racial and Ethnic Groups, n.d).

	Combined	2022	2023
Factor	n=156	n=76	n=80
Enrollment start date		2/1/2022	2/1/2023
Enrollment end date		3/6/2022	3/27/2023
Enrollment period (in days)		34	55
Enrollment day, Median (IQR)	14 (6, 28)	7 (1, 20)	20 (12, 33)
Percent of enrollment period, Median (IQR)	30.2 (15.5, 59.1)	20.6 (2.9, 58.8)	36.4 (21.8, 59.1)
Gender, n (%)			
Female	126 (80.8)	63 (82.9)	63 (78.8)
Male	27 (17.3)	13 (17.1)	14 (17.5)
Unknown	3 (1.9)	0 (0.0)	3 (3.8)
Hispanic, n (%)			
Yes	16 (10.3)	8 (10.5)	8 (10.0)
No	138 (88.5)	68 (89.5)	70 (87.5)
Unknown	2 (1.3)	0 (0.0)	2 (2.5)
Race,* n (%)			
White	50 (32.1)	17 (22.4)	33 (41.3)
African American	25 (16.0)	14 (18.4)	11 (13.8)
Asian/Asian American	54 (34.6)	27 (35.5)	27 (33.8)
Middle Eastern	6 (3.8)	5 (6.6)	1 (1.3)
Mixed	12 (7.7)	9 (11.8)	3 (3.8)
My race is not represented	4 (2.6)	3 (3.9)	1 (1.3)
Prefer not to say/Missing	5 (3.2)	1 (1.3)	4 (5.0)
Underrepresented minority,** n (%)			
Yes	44 (28.2)	27 (35.5)	17 (21.3)
No	106 (67.9)	47 (61.8)	59 (73.8)
Unknown	6 (3.8)	2 (2.6)	4 (5.0)
1st generation, n (%)			
Yes	47 (30.1)	28 (36.8)	19 (23.8)
No	107 (68.6)	46 (60.5)	61 (76.3)
Unknown	2 (1.3)	2 (2.6)	0 (0.0)
GPA, mean (SD)	3.7 (0.3)	3.6 (0.3)	3.7 (0.4)
School year, n (%)			
Four-year program	98 (62.8)	52 (68.4)	46 (57.5)
Two-year Community College	5 (3.2)	3 (3.9)	2 (2.5)
Medical School	8 (5.1)	6 (7.9)	2 (2.5)
Graduate School	32 (20.5)	9 (11.8)	23 (28.7)
Post-Baccalaureate	7 (4.5)	6 (7.9)	1 (1.3)
Not Enrolled	6 (3.8)	0 (0.0)	6 (7.5)

Time to application completion 2019-2023 

Across all years (2,494 applicants), the application period ranged from 75 - 89 days and median time to application was 71 days (IQR: 57, 76) after application period opened, reflecting 88.2% (IQR: 73.0, 97.3) of the total application period (Table 1). 

Race and Ethnicity Differences

**Figure 1:**
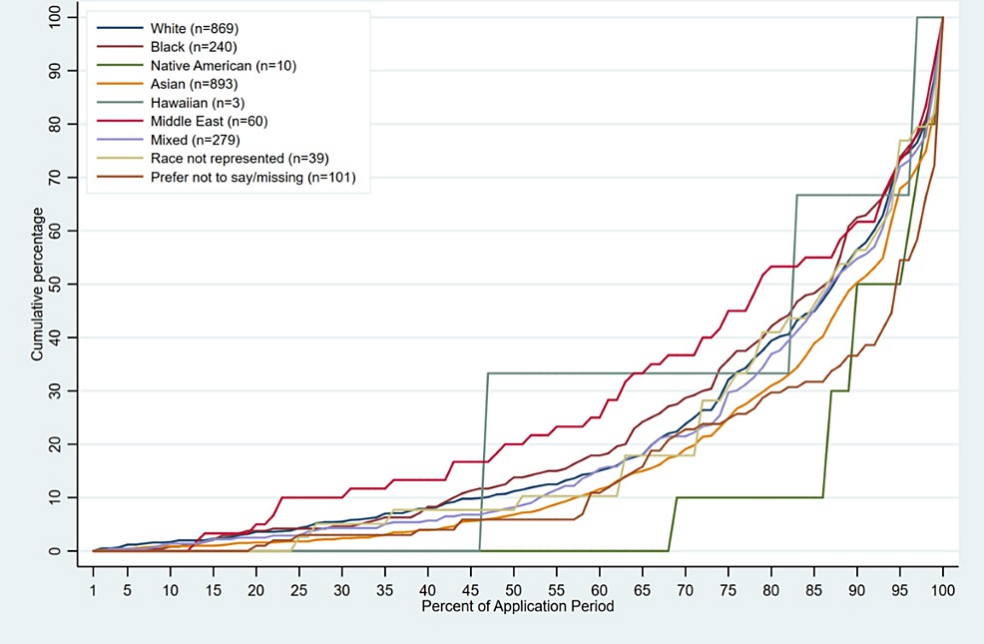
Timing of Completion in Application (Hybrid Summer Research Training) Program During the Application Period by Race (2019 –2023; n =2494).

**Figure 2:**
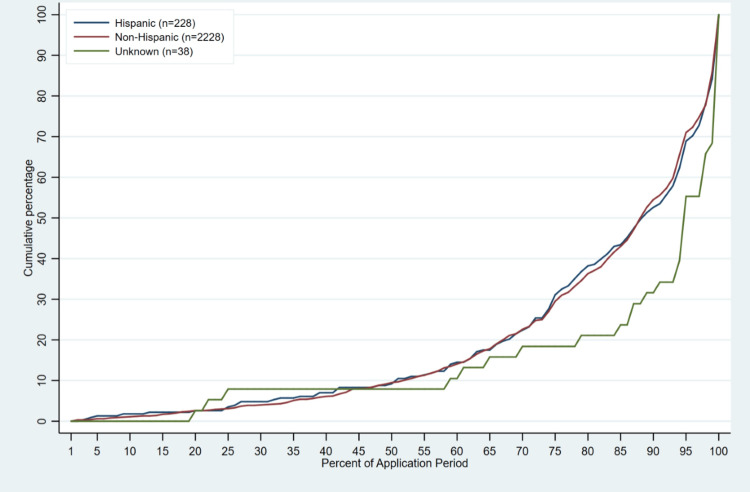
Timing of Application Completion in Application Program During the Application Period by Ethnicity (2019 –2023; n=2494).

Median time to application completion across all race groups ranged from 66 - 74 days. Applicants who self-identified as Middle Eastern (median application day = 66 [IQR: 47, 76]; 78.8% [IQR: 59.5, 95.8] of application period completed) and Native Hawaiian/Other Pacific Islander (median application day=66 [IQR: 41, 86]; 82.5% [IQR: 46.1, 96.6] of application period completed), on average, applied to the program before all other groups, followed by African American, White, and “My Race is Not Represented” applicants (Table 3, Figure 1). Applicants who preferred to not report race (median application day =74 [IQR: 62, 76]) and applicants who self-identified as Native American (median application day =73 [IQR: 66, 76]) and Asian/Asian American (median application day =73 [IQR: 62, 76]) applied to the program last on average compared to other race groups (Table 3, Figure 1). There were no differences in time to application between Hispanic and non-Hispanic students (Table 3, Figure 2). 

**Table 3:** Comparison of Application (Hybrid Summer Research Application) and Enrollment Program (Virtual Asynchronous Enrollment) Participant Characteristics by Race and Ethnicity (2019 to 2023). *Mutually exclusive race categories.

	Application Program			Enrollment Program		
Factor	n	Apply day, median (IQR)	Percent of application period, median (IQR)	n	Enroll day, median (IQR)	Percent of enrollment period, median (IQR)
Race*						
White	869	70 (56, 75)	87.6 (71.1, 96.1)	50	16 (10, 29)	31.6 (20.6, 56.4)
African American	240	70 (55, 75)	86.3 (66.0, 96.0)	25	13 (10, 23)	29.4 (18.2, 67.6)
Native American	10	73 (66, 76)	92.8 (86.8, 97.8)	0	---	---
Asian/Asian American	893	73 (62, 76)	90.0 (75.3, 98.7)	54	11 (2, 25)	20.3 (5.9, 55.9)
Native Hawaiian/Other Pacific Islander	3	66 (41, 86)	82.5 (46.1, 96.6)	0	---	---
Middle Eastern	60	66 (47, 76)	78.8 (59.5, 95.8)	6	3 (1, 15)	7.4 (2.9, 27.3)
Mixed	279	71 (56, 75)	86.8 (73.7, 96.6)	12	28 (19, 28)	75.5 (55.9, 82.4)
My race is not represented	39	70 (57, 76)	86.7 (72.0, 95.0)	4	9 (5, 12)	19.9 (9.7, 33.8)
Prefer not to say/Missing	101	74 (62, 76)	95.0 (75.3, 100)	5	22 (22, 31)	40.0 (40, 56.4)
Hispanic						
Yes	228	71 (56, 76)	88.5 (72.0, 97.3)	16	10 (6, 17)	18.2 (11.1, 45.6)
No	2,228	71 (57, 75)	88.2 (72.8, 97.3)	138	15 (5, 28)	30.9 (14.7, 64.7)
Unknown	38	76 (68, 76)	95.0 (86.3, 100)	2	22 (22, 22)	40.0 (40.0, 40.0)

Time to enrollment completion 2022-2023 

Across both years (156 enrollees), the enrollment period ranged from 34 - 55 days and median time to enrollment was 14 days (IQR: 6, 28) after the enrollment period opened, reflecting 30.2% (IQR: 15.5, 59.1) of the total enrollment period completed. In comparison to 2022, students who enrolled in 2023 enrolled later in the enrollment period (median time to enroll was seven days [IQR: 1, 20] in 2022 vs 20 days [IQR: 12, 33] in 2023, respectively; Table 2). 

Race and Ethnicity Differences

**Figure 3:**
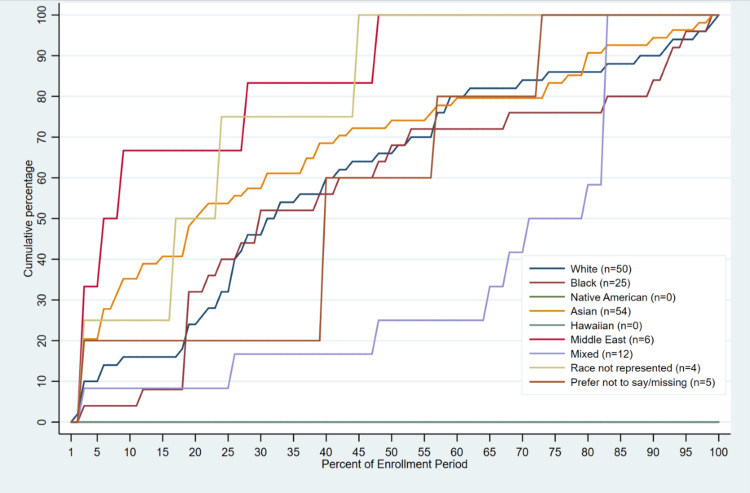
Timing of Enrollment Completion in Virtual Asynchronous Enrollment Certificate Program During the Enrollment Period by Race (2022 –2023; n=156).

**Figure 4:**
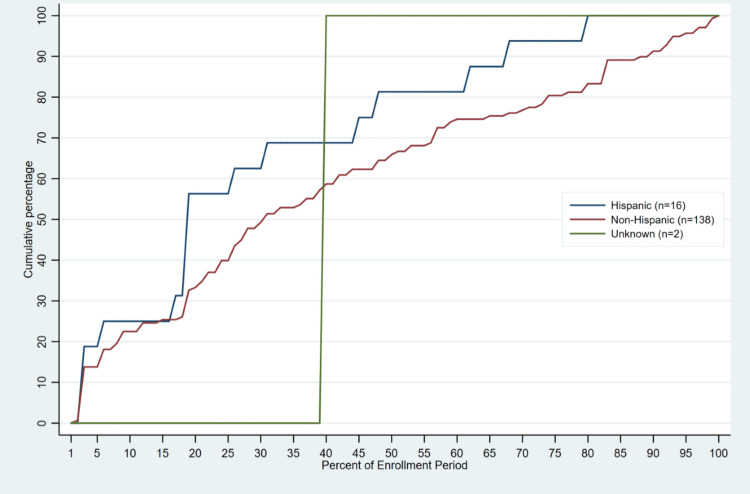
Timing of Enrollment Completion in Virtual Asynchronous Enrollment Certificate Program During the Enrollment Period by Ethnicity (2022 –2023; n=156).

Median time to enrollment ranged from 3-22 days across all race groups. On average, students who self-identified as Middle Eastern enrolled in the program before other race groups (median enrollment day = 3 [IQR: 1, 15]; 7.4% [IQR: 2.9, 27.3] of enrollment period completed), followed by “My Race is Not Represented”, Asian/Asian American, African American, and White students (Table 3, Figure 3). Students who self-identified as Mixed, on average, enrolled in the program last (median enrollment day = 28 [IQR: 19, 28]; 75.5% [IQR: 55.9, 82.4] of enrollment period completed). Hispanic students enrolled in the program earlier than non-Hispanic students (median enroll day = 10 [IQR: 6, 17] vs 15 [IQR: 5, 28]; Table 3, Figure 4). 

Comparison of program by race and ethnicity

Applicants were 9.6% and 6.4% Black (application vs enrollment programs; p<0.0001), 0.4% and 0% Native American to the application and enrollment programs, and 9.1% and 10.3% Hispanic (application vs enrollment programs; p=0.6).

## Discussion

Racial and ethnic diversity in higher education is urgently needed to increase diversity in medicine and research so that a more representative healthcare workforce [25,26] can contribute to improving population health [27]. However, while higher education mission statements often claim diversity in gender identity, race, ethnicity, socioeconomic status and diversity of thought as goals [28,29], there is little information on how racial and ethnic differences in timing of academic program application or enrollment completion may affect attaining these goals. Today’s ongoing underrepresentation of a more diverse STEM workforce, despite a plethora of educational enrichment programs compels a more comprehensive approach to elucidating not only the strengths and shortcomings of these programs but the application process for participation [30]. The purpose of this study was to examine differences among racial and ethnic groups across two higher education enrichment program types through timing of student application and enrollment completion. The main findings of this study of two injury and violence prevention programs are: 1) Applicants were 9.6% and 6.4% Black (application vs enrollment programs; p<0.0001), 0.4% and 0% Native American to the application and enrollment programs, and 9.1% and 10.3% Hispanic (application vs enrollment programs; p=0.6), 2) Across all racial and ethnic groups, students applied later (last 15% percent of application period) in the competitive application program than to the first-come first-served enrollment program in which students enrolled throughout the enrollment period, and 3) Across both program types, there were racial and ethnic differences in time to application and enrollment start and completion. 

Our findings that time to application and enrollment completion in both academic enrichment programs varied by race and ethnicity is similar to observations from college admissions trends [31-33], which show that minoritized racial groups and students from lower socioeconomic backgrounds tend to apply later and/or fail to complete certain sections of the application when compared to White students and students from higher socioeconomic backgrounds. In this study, while most students applied to the application program late in the application period, with 15% of the application period remaining regardless of race and ethnicity, time to application completion was later for some racial and ethnic groups, specifically, Asian/Asian American, Native Americans, and individuals who preferred not to report their race applied the latest of the other races. Regarding the enrollment program, students who identified as mixed-race were last to apply and Native American students were not represented in the enrollment program at all. 

Like college admissions, the observed differences in time to application in our application program may reflect differences in academic preparation, support, and/or time. Research demonstrates that underserved students are less likely to consider early decision (ED) application to colleges [34-37]. Reasons include inadequate financial capital to prepare for standardized exams and the binding nature of ED without a clear commitment of financial aid, as well as a lack of cultural capital, reflecting less knowledge and experience from their support networks (parents, counselors, social networks) that decrease awareness of educational opportunities [38]. Another study examining undergraduate admissions showed that earlier applicants were more likely to be accepted by preferred school choices and that students who apply later in the application cycle may be at a disadvantage [38], thereby explaining the small numbers of Native American students in the final cohort of our application program. 

Unlike our application program, enrollment program submissions occurred throughout the open enrollment period. While rolling admissions affords applicants more flexibility, literature on the implications of rolling admissions is sparse. One study conducted by Aalboe and colleagues [39] found African American students in dental school were more likely to apply later in the rolling admission timeline, which resulted in higher competition for a smaller number of positions. Although our enrollment program was provided at no cost and asynchronous which offered a flexible educational experience, some groups enrolled later than other groups (e.g., Middle Eastern students enrolled earlier than other racial groups and students who identified as Mixed race enrolled in the program last). Native American students were missing entirely in our enrollment enrichment program and poorly represented in the application enrichment program. Similar to the application program, our enrollment program findings may reflect financial capital and cultural capital disparities. Another more recently explored societal mechanism that helps to perpetuate inequality in education is opportunity hoarding - “when ‘members of a categorically bounded network acquire access to a resource that is valuable, renewable, subject to monopoly’ and exclude other groups [Native Americans in the application group] from access to it” [40]. The increased representation of Whites (34.8%) and Asians (35.8%) in our application program compared to URM (21.9%) compels consideration of the influence of this factor in our final cohort. The development of free educational opportunities as a standalone incentive to promote equity in educational attainment may be insufficient to reduce educational access disparities. This is evident in our work showing that stipends are needed to help promote Black representation and that free enrollment programs do not incentivize Native American student participation.

Academic enrichment programs can prepare students for and supplement higher education [22] and our results suggest a need to better understand how application processes affect access to academic enrichment programs and to develop application and enrollment processes to improve access in order to help mitigate educational attainment disparities such as educational effectiveness (quality of school, resource availability, student GPA). Strategies may also need to consider other aspects of learners’ experiences like imposter syndrome, which disproportionality affects women and underrepresented minoritized groups and may compel some to delay applying or not apply to enrichment programs due to feelings of unpreparedness for success [41]. Enrolling in institutions with a larger number of students, counsellors and instructors with shared racial identity may afford racially minoritized students important connections for building social capital integral for gaining insights, knowledge, and resources needed to navigate a system they themselves may be unfamiliar with for a variety of reasons (i.e., being a first-generation college student [42]). 

Implications and future directions 

This work provides new insights into application and enrollment patterns for higher academic enrichment programs by race and ethnicity and suggests that additional strategies are needed to support applications and enrollment of students underrepresented in healthcare and science to meet the needs of a diverse healthcare population. Although not directly addressing academic enrichment programs, debates on how to achieve racial equity in higher education admissions have been contested since the late 1970’s when the Supreme Court reached a split decision in the Regents of the University of California v. Bakke case, a court case centered on the legality of higher education programs (i.e., law schools) considering race as a criteria for admission [43]. Although several states have removed race-based affirmative action from their college admissions practices altogether starting in the 1990’s [44,45], scholars have highlighted the importance of racial/ethnic diversity on college campuses [46,47]. Equally important is attention to assuring equitable access to academic enrichment programs such as ours to help promote continued support of these students. Future work is needed to examine racial and ethnic differences in context with other important intersectional identities and factors such as gender, socioeconomic status, and parent education. Educational research is also needed to develop research methods to uncover nuances within application and enrollment educational processes [32,35]. Further, mixed methods and longitudinal research are needed to examine the long-term impacts of undergraduate students who participate in higher education enrichment programs by race, ethnicity, and intersectionality as well as reasons for why students choose not to disclose this information. 

Strengths and limitations 

This study has some strengths and limitations. Main strengths are that this work contributes to the gap in knowledge in higher education research as to how higher education enrichment program (application or enrollment) type may affect access to education for racial and ethnic groups [48,49], we utilized application data over multiple years to capture a longitudinal trend in higher education internship admission practices, and we provide new information on the ability of program type to promote Black student participation. Limitations are that we did not collect information on socioeconomic status, details on mixed race, or mentorship (e.g., a student working with a professor who encouraged an application) or other enabling factors which may affect application or enrollment timing and disparities [31,32,50]. We did not gauge interest in program type or change in program type during the COVID-19 pandemic, there were few (application program) or none (enrollment program) participants who self-identified as Native Americans which is why we did not examine underrepresented group data, and the number of years of available data varied by program type. 

## Conclusions

The competitive application program received most applications in the last 15% of the application period (median time to application 71 [IQR 57,76]) days and students enrolled in the enrollment program earlier and throughout the enrollment period (median time to enrollment 14 [IQR 6,28]) days. However, across both program types, underrepresented minority students completed application and enrollment procedures later and Native American students were not represented in the enrollment program. These findings suggest that free enrollment programs alone do not incentivize educational attainment for all groups, application programs with stipend promote more representation of Black applicants, and support the idea that application processes with early decisions and/or rolling admissions may not equally promote racial and ethnic diversity for all groups. Educational organizations should develop creative strategies to provide funding opportunities for students to have access to enrichment learning opportunities. 
